# Fighting Global Disparities in Cancer Care: A Surgical Oncology View

**DOI:** 10.1245/s10434-016-5194-3

**Published:** 2016-04-01

**Authors:** Harald J. Hoekstra, Theo Wobbes, Erik Heineman, Samuel Haryono, Teguh Aryandono, Charles M. Balch

**Affiliations:** Division of Surgical Oncology, University of Groningen, University Medical Center Groningen, Groningen, The Netherlands; Department of Surgery, Radboud University Medical Center, Radboud University, Nijmegen, The Netherlands; Department of Surgery, University of Groningen, University Medical Center Groningen, Groningen, The Netherlands; Association of Surgeons of the Netherlands, Utrecht, The Netherlands; Department of Surgery, National Cancer Center, Dharmais Hospital, Jakarta, Indonesia; Department of Surgery, Universitas Gadjah Mada, Yogyakarta, Indonesia; Faculty of Medicine, Universitas of Gadjah Mada, Yogyakarta, Indonesia; Division of Surgical Oncology, Department of Surgery, University of Texas Southwestern Medical Center, Dallas, TX USA

## Abstract

Cancer is the second leading cause of death globally after cardiovascular disease. Long-term cancer survival has improved in the Western world due to early detection and the use of effective combined treatment modalities, as well as the development of effective immunotherapy and drug-targeted therapy. Surgery is still the mainstay for most solid tumors; however, low- and middle-income countries are facing an increasing lack of primary surgical care for easily treatable conditions, including breast, colon, and head and neck cancers. In this paper, a surgical oncology view is presented to elaborate how the Western surgical oncologist can take part in the ‘surgical fight’ against global disparities in cancer care, and a plea is made to strive for structural solutions, such as a partnership in surgical oncology training. The pros and cons of the use of eHealth and mHealth technologies and education programs for schools and the community are discussed as these create an opportunity to reach a large portion of the population in these countries, at low cost and with high impact.

Global disparities in healthcare arise from a complex interplay of economic, educational, social, and cultural factors. At the Lancet Commission on Global Surgery 2030 meeting on 17 January 2014 in Boston, Jim King, President of the World Bank, stated that “surgery is an indivisible, indispensable part of healthcare and can help millions of people lead healthier, more productive lives.” In 2015, The Lancet Commission published an extensive report with evidence and solutions for achieving health, welfare, and economic development,^1^ while another report was published in regard to the delivery of safe, affordable, and timely cancer surgery.^2^ These two extensive papers were discussed shortly afterwards in editorials in *Annals of Surgical Oncology* and the *European Journal of Surgical Oncology*.^2^,^3^

Since 2013, cancer has been the second leading cause of death globally after cardiovascular disease, and is expected to increase in all countries due to population growth, aging, and increasing prevalence of risk factors.^4^ An estimated 20 % of ‘global surgery’ has been denoted as ‘cancer surgery’. In high-income countries (HICs), the standards of evidence-based multimodality cancer treatment (surgery, radiation, and systemic treatment) have been well defined, whereas the standards of surgical and anesthesia care in low- and middle-income countries (LMICs) are stagnating or regressing.^1^ This disparity in cancer care requires an accurate analysis to improve overall (surgical) cancer care.^5^

In this paper, the difference between cancer surgery, training, and quality assurance in HICs, and the common lack of such provisions in LMICs, are addressed. The potential role of community- and university-based surgical oncologists and surgical oncology societies in fighting the global disparity in cancer care is discussed. Finally, suggestions are made as to the use of new electronic technologies in teaching healthcare workers in LMICs and creating a general awareness of (malignant) diseases with lifestyle risk factors.

## The Evolution of Cancer Surgery, Training, and Quality Assurance

In most Western countries, long-term survival has doubled for some cancers, such as breast and colon cancer, over the past 40 years through early detection, the use of effective combined treatment modalities, radiation, and/or systemic treatment, reducing the 30-day postoperative mortality due to improved surgical techniques, surgical equipment with less intraoperative and postoperative bleeding, and, last but not least, anesthesia and intensive care facilities. Today, personalized cancer surgery has become a reality with the conservation of the integrity and function of the body and the preservation of quality of life. Compared with LMICs, surgeons in HICs have the ability to stage cancer patients using computed tomography (CT), magnetic resonance imaging (MRI), positron emission tomography (PET), and/or sentinel lymph node biopsy (SLNB), and to use effective combined treatment modalities with the various radiation techniques, treatment planning, radiation doses, and systemic approaches ranging from hormonal, chemotherapy, and immunotherapy to targeted drug therapy, as well as the various surgical interventions, from conventional surgery and laparoscopic procedures to robotic and image-guided surgery. For many types of cancer, surgery has plateaued as a treatment in regard to morbidity and mortality, local control, and long-term survival, whereas, in palliative care, the role of surgery is explored continuously. New developments are still being made in minimally invasive surgery, such as single-incision laparoscopic surgery, robot-assisted laparoscopic surgery, transanal endoscopic microsurgery, and natural orifice transluminal endoscopic surgery.^6^ All these procedures are accompanied by steep learning curves and require centralization of the surgical care of these cancer patients.

There is significant diversity in surgical (oncology) training programs and certification requirements, with an inverse correlation to a country’s income. Almost all HIC and LMIC surgical (sub)specializations are organ-based rather than focused on oncology, with significant variability in the training of surgical oncologists worldwide.^7^ Although today most LMICs do not have these structured surgical oncology training pathways, at the same time, a few LMICs have had structured surgical oncology fellowships in place for a number of decades now. In HICs, surgeons have taken the lead with regard to further specializing in cancer surgery at high-volume centers, with centralization of cancer surgery in accredited cancer centers.

Thirty-five national surgical oncology societies currently comprise the World Federation of Surgical Oncology Societies (WFSOS), but no international, equally acknowledged surgical oncology curriculum exists.^8^ The Society of Surgical Oncology (SSO) and European Society of Surgical Oncology (ESSO) have well-defined surgical oncology curricula and, in Mexico, a program with certification was recently started under the auspices of the Consejo Mexicano de Oncología (CMO).^9^–^11^ Training programs are also running in other LMICs, but a great diversity exists globally, and training and certification should therefore be streamlined.^7^ In Europe, the Union Européenne des Médecins Spécialiste (UEMS) represents more than 50 medical disciplines in 34 countries, and the Division of Surgical Oncology was established in 2003 to promote excellence in cancer surgery across Europe, along with European board examinations and certifications.^12^ Board certification for cancer surgery was introduced in the USA and The Netherlands in 2011 and 2014, respectively.^13^,^14^

These board certifications and quality assurance programs improved the surgical oncology outcome in HICs, but LMICs still face the burden and need for more surgical care. The incidence of cancer has increased tremendously in LMICs, leading to a growing discrepancy between the demands and opportunities of surgical care. Too few surgeons are trained in basic surgical oncological procedures.^15^

## Burden of Surgical Cancer Care

Advances in (cancer) surgery have been ignored in LMICs. Well-equipped hospitals are present in most capitals of LMICs, but a majority of the surgeons work with limited resources and a majority of their cancer patients are diagnosed at an advanced stage, with limited opportunities for effective cancer treatment. Even if patients in LMICs are treated, they are at high risk of recurrence and disease-related morbidity and mortality. The financial condition of each patient and financial support from family are generally limited as adequate health insurance is obsolete and bureaucratic. With the exception of the big cities, limited advanced diagnostic radiology capabilities are available in LMICs and, in general, there is a lack of (updated) radiation facilities. Furthermore, the Lancet Oncology Commission suggests that investment in and expansion of global access to radiotherapy could save lives and may have positive economic benefits; however, first and foremost, this requires billions of dollars of investments. Second, it also requires education and licensing programs for LMIC physicians specializing in radiation oncology at foreign cancer institutions in HICs.^16^ Systemic cancer treatment has been inhibited by the lack of well-equipped pharmacies, and effective chemotherapy protocols with targeted therapy are generally not affordable. Complications associated with systemic treatment may add to the burden of care due to the lack of effective antibiotics and/or injectable growth factors. The opportunities that most HIC surgeons have for maintaining the integrity of the patient’s body are not available in LMICs, especially when the patients have advanced cancer. The gap between the healthcare system in HICs and LMICs is not expected to close soon.

Because surgeons are the primary caregivers for cancer in LMICs, competent surgeons should be trained in a shorter period of time to become ‘basic surgical oncologists’ for the most commonly diagnosed cancers. This training should also include the basic principles of systemic anticancer treatment, as well as palliative care. How can Western surgeons and (surgical) oncologists play a major role in addressing and tackling the burden of surgical cancer care with disproportionate increases in that burden in LMICs? To achieve this role there are, among others, some important conditions to be fulfilled: (i) acknowledge the global surgical oncology disease burden; (ii) commence a global movement through the World Health Organization (WHO), making efficient use of the International Agency for Research on Cancer (IARC) programs, the US National Cancer Institute Center for Global Health, the Lancet Global Surgery Commission, and global cancer consortiums (GCC); (iii) train surgeons to be surgical oncologists through partnership programs with foreign institutions and/or through collaboration with international (surgical) cancer society programs focusing on LMICs, or through Comprehensive Cancer Centers; and (iv) HICs can be involved in cancer research in LMICs.^5^,^17^–^20^ Qualitative and quantitative research should be encouraged in LMICs to support decision making and patient access to healthcare facilities, or to obtain data to make policy makers in LMICs aware of the effects of the collaborations.^21^ Implementation of these points is an important starting point to provide a structural solution to the anticipated lack of knowledge and practical capabilities in LMICs, rather than the present practice of Western surgeons temporarily providing voluntary support to surgical clinics individually or through welfare organizations. The goal should be to create a situation in LMICs, as summarized in the following proverb: “Give a man a fish and you feed him for a day; teach a man to fish and you feed him for a lifetime.”

## Opportunities for Education Through Collaboration

The WHO has just launched the Global Cancer Country Profiles, which include reference data on cancer mortality and incidence, risk factors, availability of cancer country plans, monitoring and surveillance, primary prevention policies, screening, treatment, and palliative care.^22^ Programs are related to cancer control, prevention, early detection, and palliative care. National smoking bans and programs promoting obesity rate reduction through lifestyle changes will have a dramatic effect on decreasing the incidence of cancer, cardiovascular diseases, and type II diabetes in HICs and LMICs. More than 168 countries have signed the WHO Framework Convention on Tobacco Control.^23^ In The Netherlands, for example, an initiative has been launched by physicians and healthcare entrepreneurs to legislate a ban on tobacco purchase and use by any individual born from 2016 onwards. Even smokers agree with this ban for their future children. The WHO also has a global nutrition program with the implementation of the Global database on the Implementation of Nutrition Action (GINA).^24^ In both programs, HICs can support LMICs in achieving the goals of national campaigns to reduce smoking and decrease the overweight population. The Western world can support these campaigns with the development of specific social media campaigns (see the eHealth and mHealth section).

In a recent paper, Brennan discussed the possibility of sophisticated third-year clinical rotation fellowships within a community cancer program for residents from LMICs.^5^ Regular postgraduate (surgical) cancer courses in LMICs will eventually have an impact on the provision of healthcare. Surgical oncologists or their institution may adopt the program or collaborate with an overseas university or regional hospital and start, through a memorandum of understanding, a collaborative clinical surgical oncology/research initiative. Over several decades, more than 20 Indonesian surgeons, with the support of the Department of Surgery of the University Medical Center Groningen (UMCG) and Dutch Cancer Society, received specific training in the basic principles of surgical (oncologic) procedures and palliative care at the Department of Surgical Oncology, Department of Urology, Department of Neurosurgery, and Department of Plastic Surgery of the UMCG. Some received board certifications prior to their return to Indonesia, but they all later became surgical (oncology) leaders in Indonesia and distributed their knowledge through their institutional surgical training programs and surgical societies (e.g. oncology societies such as Perhimpunan Ahli Bedah Onkologi Indonesia; PERABOI, the Indonesian Society of Surgical Oncology). Recently, a similar residents training program was initiated between the Department of Surgery of the UMCG and the Department of Surgery of the University Hospital Paramaribo in Suriname. This UMCG training program can easily be copied by other institutions. Another possibility is to start a foundation with the goal of improving (surgical) cancer care in a hospital, region, country, or continent.^25^

What are the opportunities for the two largest surgical oncology societies in the fight against cancer, nationally and globally?^9^,^10^ The SSO has 2721 members (52 members in LMICs, 2 %) and publishes the *Annals of Surgical Oncology* (impact factor [IF] 3.943), while the ESSO has 3822 members (49 members in LMICs, 1 %) and publishes the *European Journal of Surgical Oncology* (IF 3.009). Both societies have reduced their fees for membership and annual meetings for participants from LMICs. Why are they still unable to grant free electronic subscriptions for their journals to surgeons, residents, and medical students in LMICs? This is the way both societies could support our current and future colleagues in LMICs as the time of studying from old-fashioned textbooks is over.

## eHEALTH and mHEALTH

An important instrument for collaboration through education is available today, and there is an opportunity to use modern electronic technology in teaching medical students, physicians, specialists, healthcare workers, and nurses, as well as in providing healthcare. eHealth is the application of information and communication technology in the service of health. Mobile health (mHealth) is the practice of medicine and public health supported by mobile devices. The boost from high-speed internet on smartphones and tablets has contributed to the rapid development of eHealth and mHealth technology, such as Short Message Service (SMS), Multimedia Message Service (MMS), telemonitoring, telecoaching, telecare, teleconsultation, telediagnosis, teleradiology, telesurgery (robotic surgery), teleconferencing (regionally, nationally, internationally) and e-consults. Built-in cameras and video recorders can be used as sensors to measure or track vital signs, such as heart rate and respiration. Today, we have great variation in medical tricorders, portable handheld scanning devices used by consumers, patients, caregivers, nurses, or physicians to (self)diagnose medical conditions within percentages, or show and summarize a person’s health status, and the data can be electronically transferred to anywhere in the world, even from rural areas. FaceTime provides the possibility of rapidly exchanging information between clinicians, such as when facing an urgent intraoperative problem. For quick communication among staff members, WhatsApp is available, a multimedia smartphone application for patient care and academic endorsement. There are no boundaries or disparities with eHealth and mHealth technology.

The implementation of these techniques in LMICs will not be without difficulties. The application of eHealth facilities may be distinguished two ways: support of primary healthcare professionals, including medical students and residents, or education for people living in rural and remote areas. Most LMICs currently have an extensive cellular phone network and availability of Internet technology, but they are facing the challenge of receiving continuous high-quality, affordable, and universally accessible health education. The application of eHealth facilities for both purposes may be limited through a variety of problems. In rural and remote areas in certain parts of the world, these technologies are probably not working as optimally as predicted. Although technological problems may have solutions, there are human factors, such as behavioral changes, system constraints, and privacy limitations, which may set barriers in efforts to provide adequate healthcare information and administer care.^26^ A recent Cochrane analysis of studies on enhancing the effects of primary health interventions by mobile phone applications, such as SMS and MMS, demonstrated that much is not yet known about the long-term effects or potential negative consequences, although one study showed short-term effects of smoking cessation.^27^ A study on the effects of a telehealth system in rural and remote areas of Brazil indicated that most of the professionals were satisfied and that the system was cost effective and led to access to specialized healthcare. The main lessons learned from this study were that the system requires a broad collaborative network, must be simple, should have face-to-face components, and should be applied to address the problems for which there is a high service demand; however, the long-term effects of the system require further study.^28^ In a recent systematic review of the implementation of mobile communication techniques in Africa, the conclusion was that these techniques pose a potential to becoming an important part of the health sector to establish innovative approaches to the delivery of care. The benefits have also been highly recommended, but it is clear that the projects are not a solution to the challenges that health systems face in many African countries. More evidence-based research is necessary in the field of mobile communication technique implementation, especially on a large scale and for a long time.^29^ The potential of eHealth and mHealth technology in education towards cancer prevention, diagnosis and treatment, and the (surgical) fight against cancer in LMICs, have not been properly investigated.

## Prospects and Suggestions

No major changes are foreseen in the current global disparities in cancer care or global gross domestic products. Future world population growth will come from the LMICs, i.e. countries with a high economic vulnerability, low life expectancy at birth, low per capita income, low levels of education, and negative effects of urbanization. These countries will also have a tremendous increase in cancer burden by a limited healthcare system. In 2030, 70 % of all cancer deaths will occur in LMICs, and there will be a shift in the distribution of all types of cancer due to increased cancer incidence rates with non-infectious etiology via Western lifestyle changes attributable to economic development. The ratio of cancer incidence to mortality is low in HICs (46 %) and high in LMICs (75 %);^30^ however, there have been developments that give us a reason to be optimistic. On 4 February 2015, the Health Minister of the Republic of Indonesia, Nila Farid Moeloek, decreed the Commitment to Cancer Management in Indonesia. One of the commitments was to support public regulation for prevention via a healthy lifestyle against cancer.^31^ Although this is a good national initiative, the impact on overall (surgical) cancer care and oncological outcome will be limited.

To start with international collaboration between countries and societies through the WHO, IARC, US National Cancer Institute Center for Global Health, the Lancet Global Surgery Commission, GCC, or surgical oncology societies (e.g. SSO^2^ and ESSO^3^) might be the first step on the way to fighting this cancer burden by way of (i) *educational exchange programs* to enhance the curriculum content of undergraduate, postgraduate, surgical resident, and fellowship training programs in the basic principles of surgical oncology and multidisciplinary cancer conferences, as well as virtual training; (ii) the implementation of e*Health and mHealth technology and education programs* at schools and to the general public, for education in health behavior, cancer prevention, diagnosis, and (surgical) cancer treatment; and (iii) *simple tricorders* for cancer diagnosis with mHealth technology.

Digital technology is transforming health and social care in the HICs, but also expanding to LMICs.^32^ The Department of Health and Human Services is providing funding opportunities through the National Cancer Institute RFA-CA-15-024 Cancer Detection, Diagnosis, and Treatment Technologies for Global Health, e.g. LMICs, the UG3/UH3 mechanism.^33^ Education with eHealth and mHealth technology, as well as collaboration and support from community and university-based surgical leaders and surgical oncology societies, is essential to successfully decrease the global cancer burden. According to Murray Brennan, ‘Western surgical oncology clinics’ can provide life-changing experiences for surgical residents to achieve a better understanding of cancer and the surgical options for fighting cancer through an educational exchange instead of a sophisticated fellowship.^5^
